# Maternal obesity and its effect on labour duration in nulliparous women: a retrospective observational cohort study

**DOI:** 10.1186/s12884-017-1413-6

**Published:** 2017-07-12

**Authors:** Karen Louise Ellekjaer, Thomas Bergholt, Ellen Løkkegaard

**Affiliations:** 1 Department of Obstetrics and Gynaecology, Nordsjællands Hospital, University of Copenhagen, Dyrehavevej 29, 3400 Hillerød, Denmark; 2 Department of Obstetrics and Gynaecology, Rigshospitalet, University of Copenhagen, Blegdamsvej 9, 2100 Copenhagen, Denmark

## Abstract

**Background:**

Obesity is increasing among primipara women. We aimed to describe the association between body mass index (BMI) during early-pregnancy and duration of labour in nulliparous women.

**Methods:**

Retrospective observational cohort study of 1885 nulliparous women with a single cephalic presentation from 37 0/7 to 42 6/7 weeks of completed gestation and spontaneous or induced labour at Nordsjællands Hospital, University of Copenhagen, Denmark, in 2011 and 2012.

Total duration of labour and the first and second stages of labour were compared between early-pregnancy normal-weight (BMI <25 kg/m^2^), overweight (BMI 25–29.9 kg/m^2^), and obese (BMI ≥30 kg/m^2^) women. Proportional hazards and multiple logistic regression models were applied.

**Results:**

Early pregnancy BMI classified 1246 (66.1%) women as normal weight, 350 (18.6%) as overweight and 203 (10.8%) as obese. No difference in the duration of total or first stage of active labour was found for overweight (adjusted HR = 1.01, 95% CI 0.88–1.16) or obese (adjusted HR = 1.07, 95% CI 0.90–1.28) compared to normal weight women. Median active labour duration was 5.83 h for normal weight, 6.08 h for overweight and 5.90 h for obese women.

The risk of caesarean delivery increased significantly for overweight and obese compared to normal weight women (odds ratios (OR) 1.62; 95%CI 1.18–2.22 and 1.76; 95%CI 1.20–2.58, respectively). Caesarean deliveries were performed earlier in labour in obese than normal-weight women (HR = 1.80, 95%CI 1.28–2.54).

**Conclusion:**

BMI had no significant effect on total duration of active labour. Risk of caesarean delivery increased with increasing BMI. Caesarean deliveries are undertaken earlier in obese women compared to normal weight women following the onset of active labour, shortening the total duration of active labour.

## Background

Average body mass index (BMI) has increased over the past 30 years and obesity has become a global health issue [1, 2]. This tendency has a wide range of implications in the field of obstetrics as women with a higher BMI are at risk of various complications during pregnancy such as gestational diabetes, preeclampsia, macrosomia, dystocia, and stillbirths [3, 4]. Furthermore, increasing BMI is associated with an increased rate of caesarean delivery due in part to failure of labour-progression [5–7].

Previously published studies have not clarified the extent to which BMI influences the duration of labour. One study showed no significant effect [8] whereas others describe prolonged duration of labour with increasing BMI [9–11]. It is pertinent to consider the effect of maternal BMI on the progress and duration of labour in order to facilitate decision-making on potential obstetric interventions. In order to ensure proper labour management, perceptions about labour progression in obese women should be evidence-based and not guided by assumptions.

The objective of this study was to describe the association between BMI during early-pregnancy and the duration of labour.

## Methods

The study population included women who gave birth at the Department of Gynaecology and Obstetrics, Nordsjællands Hospital, University of Copenhagen, Denmark, between January 1, 2011 and December 31, 2012.

Data were obtained through electronically based hospital charts (Doculive, DLEPR v. 4.9.3.0) describing all prenatal obstetric consultations and ultrasound scans as well as labour, delivery, and the postnatal period. Data were listed according to the Danish 10-digit personal identification number (dd-mm-yy-xxxx).

Data was extracted for women allocated to group 1 or 2a of Robsons 10 group classification system [12]. Hence, the study population included all nulliparous women with a single cephalic presentation from 37 0/7 to 42 6/7 weeks of completed gestation who were either induced or entered labour spontaneously. Multiparous women, multiple pregnancies, pre-term pregnancies, elective caesarean deliveries as well as breeches, transverse- or oblique presentations were excluded. Standard protocol for labour induction in our institution comprised prostaglandin-induced cervical ripening or induction with oxytocin. No transcervical catheters were used for induction of labour.

Maternal first trimester height and weight was recorded at the first prenatal consultation. Early pregnancy BMI was calculated and the women were grouped in BMI categories of <25 kg/m^2^, 25–29.9 kg/m^2^, and ≥30 kg/m^2^.

Gestational age was assessed with a dating ultrasound scan. Maternal age was calculated from the woman’s date of birth. Weight of the baby was recorded after birth. Hypertensive disease was grouped to reflect all women who had hypertensive diseases prior to pregnancy or who developed hypertension or preeclampsia during pregnancy. Manual chart review provided information on smoking status, labour induction, use of cardiotocography (CTG), augmentation using oxytocin, and epidural analgesia.

The primary outcome was duration of active labour. This was recorded as the total duration then subdivided into phases. Total duration of labour was calculated from the beginning of active labour until time of delivery. The beginning of active labour was defined as the onset of regular contractions combined with a dilated cervical orificium >3 cm. Women who had a cervical orificium dilated further upon arrival at the labour ward were defined as being in active labour from the time of admittance.

The total duration of labour was subdivided into phases 1 and 2. Phase 1 was defined as the period from active labour until the cervix was fully dilated, while phase 2 was defined as the remaining time from full dilatation until birth.

The secondary outcome comprised route of delivery, maternal post-partum complications, neonatal morbidity, and neonatal mortality. Apart from vaginal and caesarean delivery, mode of delivery included the level of urgency for caesarean deliveries. Level 1 required delivery within 15 min compared with 30 min for level 2 and 60 min for level 3. Maternal post-partum complications comprised post-partum haemorrhage (PPH) with bleeding in excess of 1000 ml. A 1000 ml limit for PPH was chosen from a clinical perspective as local protocol requires that women with PPH > 1000 ml should be referred to an operating theatre in case of a potential need of anaesthesia which is associated with higher risk among obese women. Foetal outcome was assessed by Apgar score at 5 min, arterial cord pH, and admission to the neonatal intensive care unit (NICU).

The study was approved by the Danish Data Protection Agency (case number: HIH-2013-012, I-Suite no: 02208).

### Statistical analysis

Baseline characteristics between the three categories of BMI (<25 kg/m^2^, 25–29.9 kg/m^2^, and ≥30 kg/m^2^) during early-pregnancy were compared using Chi-squared test. Cox proportional hazards regression was used for analyses regarding duration of labour. Models were fitted with two separate outcome measures. Firstly, an event was defined as vaginal delivery, whereas caesarean delivery was censored. Secondly, an event was defined as caesarean delivery, whereby vaginal birth was the censoring criteria.

Univariate Cox-regression models were performed. In multivariate analyses, adjustments were made for maternal height and age, birth weight, epidural analgesia, induction of labour and administration of oxytocin during labour. In our study we differentiated augmentation with oxytocin from induced labor oxytocin as follows: if oxytocin was administered before the onset of regular contractions in combination with a dilated cervical orifice >3 cm it was considered as an attempt at induction. If oxytocin was administered after the onset of regular contractions in combination with a dilated cervical orifice >3 cm it was considered as labour augmentation. As all cases were evaluated manually this reduced the risk of incorrect data due to coding errors.- All of the abovementioned factors were chosen due to their independent effect on either labour duration or rates of caesarean delivery. In addition, we wished to adjust for maternal race, however, as this information was not registered in labour charts, we were unable to extract data to include in our mutivariate analysis. Backwards elimination by stepwise regression eliminated hypertensive disease and gestational diabetes, which was therefore not accounted for in the final analysis.

Survival curves depicted the number of deliveries over time within each BMI category. The proportional hazard assumption was checked graphically. For the secondary outcome variables, univariate logistic regressions were carried out followed by multivariate analysis, with adjustment for potential confounders. *P*-values <0.05 were considered statistically significant.

The database was established using Microsoft Access 2010. Statistical analysis was performed using IBM SPSS Statistics version 19.

## Results

**Fig. 1:**
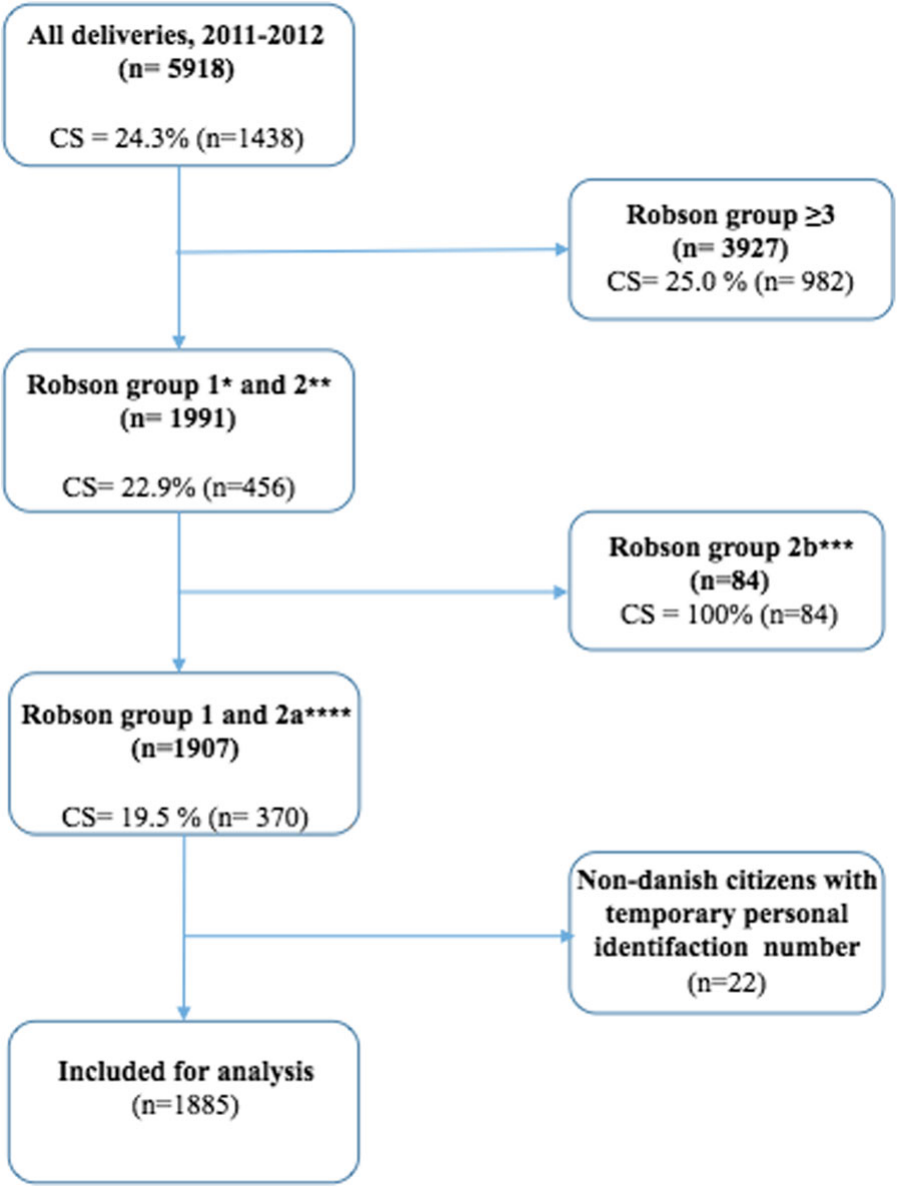
Flow chart of patients who met inclusion/exclusion criteria for the study population. *Robson group 1 comprising nullipara, singleton cephalic ≥37 weeks in spontaneous labour, ** Robson group 2 comprising nullipara, singleton cephalic ≥37 weeks who were induced or underwent caesarean before labour, *** Robson group 2a comprising nullipara, singleton cephalic ≥37 weeks who had labour induced. **** Robson group 2b; comprising nullipara, singleton cephalic ≥37 weeks who underwent caesarean section before labour

Data was extracted for a total of 1907 women. Of these, 22 non-Danish citizens were not eligible for inclusion, as they did not possess a permanent Danish personal identification number (CPR), which was necessary for comparable data. Hence, a total of 1885 women were included in the study (Fig. 1).

Women of normal weight comprised 66.1% of the population (*n* = 1246), overweight 18.6% (*n* = 350), and obese 10.8% (*n* = 203). Only 4.5% (*n* = 86) did not have BMI recorded, most often due to lack of attendance at prenatal hospital visits. BMI was equally distributed according to maternal age, height, or gestational age, whereas an association between BMI and hypertensive disease and gestational diabetes was found (*p* < 0.01) (Table 1). The proportion of induced births increased with increasing BMI, as did the use of oxytocin for augmentation of labour, epidural analgesia, and CTG monitoring. Birth weight was associated with BMI.

**Table 1:** Distribution of covariates at baseline among women in different body mass index groups

		Maternal BMI ^a^:		
		<25	25–29.9	≥30
Covariates	*p*-value	(*n* = 1246; 66.1%)	(*n* = 350; 18.6%)	(*n* = 203; 10.8%)
Maternal age	*p* = 0.92			
< 25		317 (25.4%)	86 (24.6%)	54 (26.6%)
25–30		429 (34.4%)	126 (36.0%)	74 (36.4%)
30–35		376 (30.2%)	100 (28.5%)	59 (29.1%)
> 35		124 (10.0%)	38 (10.9%)	16 (7.9%)
Height	*p* = 0.75			
< 160 cm		124 (10.0%)	36 (10.3%)	15 (7.4%)
160-169 cm		595 (47.7%)	178 (50.9%)	106 (52.2%)
170–179 cm		477 (38.3%)	124 (35.4%)	73 (36.0%)
≥ 180 cm		50 (4.0%)	12 (3.4%)	9 (4.4%)
Hypertensive disease	*p* < 0.01			
No		1132 (90.9%)	271 (77.4%)	158 (77.8%)
Yes		114 (9.1%)	79 (22.6%)	45 (22.2%)
Gestational Diabetes	*p* < 0.01			
No		1218 (97.8%)	335 (95.7%)	188 (92.6%)
Yes		28 (2.2%)	15 (4.3%)	15 (7.4%)
Gestational age	*p* = 0.27			
37–38 weeks		173 (13.9%)	46 (13.1%)	36 (17.7%)
39–40 weeks		635 (51.0%)	180 (51.4%)	98 (48.3%)
≥ 41 weeks		438 (35.1%)	124 (35.5%)	69 (34.0%)
Labour Induced	*p* < 0.01			
Yes		363 (29.1%)	131 (37.4%)	98 (48.3%)
No		831 (70.9%)	219 (62.6%)	105 (51.7%)
CTG	*p* < 0.01			
Yes		906 (72.7%)	285 (81.4%)	176 (86.7%)
No		340 (27.3%)	65 (18.6%)	27 (13.3%)
Oxytocin	*p* = 0.03			
Yes		744 (59.7%)	232 (66.3%)	135 (66.5%)
No		502 (40.3%)	118 (33.7%)	68 (33.5%)
Epidural analgesia	*p* < 0.01			
Yes		501 (40.2%)	170 (48.6%)	114 (56.2%)
No		745 (59.8%)	180 (51.4%)	89 (43.8%)
Birthweight	*p* < 0.01			
<3.0 kg		187 (15.0%)	41 (11.7%)	11 (5.5%)
3.0–3.5 kg		439 (35.3%)	111 (31.8%)	80 (39.8%)
3.5–4.0 kg		456 (36.7%)	141 (40.4%)	69 (34.3%)
>4.0 kg		162 (13.0%)	56 (16.1%)	41 (20.4%)

Values are numbers (percentages)

86 women (4.5%) did not have BMI recorded and were therefore not accounted for in analysis

Labour duration could be determined for 1808 women. Fifty-eight women (3.1%) had caesarean sections due to a failed attempt of labour induction, whereas 19 women did not have the onset of regular contractions recorded. The number of women who were already in active labour upon admittance to the hospital was equally distributed with regard to BMI (*p* = 0.09). All 1808 women with determined labour duration gave birth within 24 h of the onset of active labour. Median active labour duration was 5.83 h for normal weight, 6.08 h for overweight and 5.90 h for obese women.

**Fig. 2:**
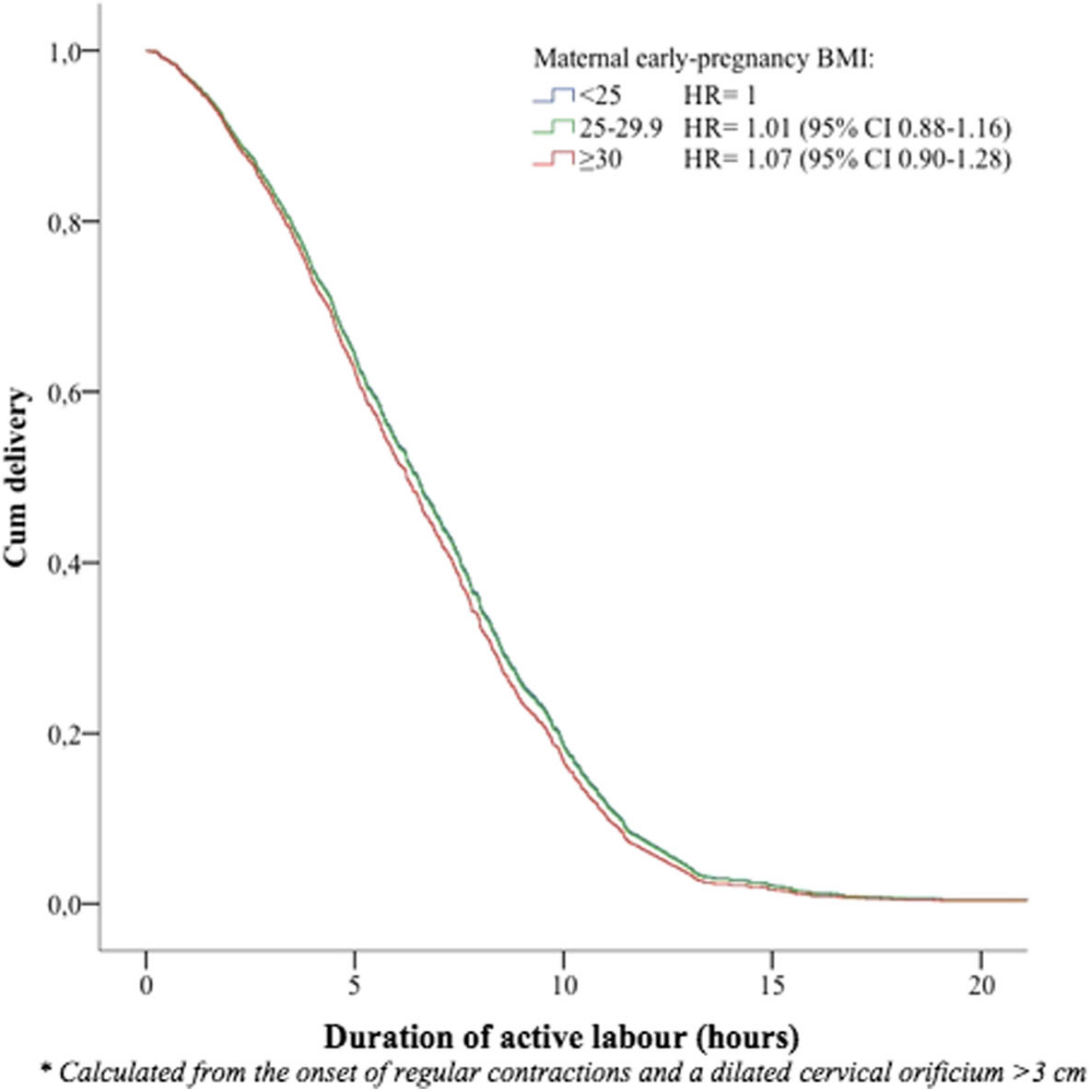
Survival plot as a function of time from onset of active labour until vaginal delivery. Event was defined as delivery. Women were censored at the time of caesarean delivery. Adjustments were made for maternal age, height, birth weight, labour induction, augmentation with oxytocin, and epidural analgesia. A hazard ratio (HR) >1 illustrates a shorter duration of labour while a HR <1 illustrates a longer duration of labour compared to the reference group consisting of normal-weight women

From univariate Cox analysis, we found a hazard ratio (HR) of vaginal delivery of 0.83 (95% confidence interval [CI] 0.73–0.96) for overweight (BMI 25–29.9 kg/m^2^) compared to normal weight women, implying a longer duration of active labour for overweight women compared with those with normal-weight (Table 2). However, after adjustment for confounders this association was not significant (HR = 1.01, 95% CI 0.88–1.16). There was no significant difference in total duration of active labour among obese women (BMI ≥30 kg/m^2^) compared to normal weight women, either before or after adjustment for confounders (Fig. 2).

**Table 2:** Cox regression showing the hazard ratio (HR) of vaginal birth in the defined body mass index (BMI) groups, with and without adjustment for covariates

	Total duration ^a^			
Covariates	Crude HR	95% CI	Adjusted HR	95% CI
BMI				
< 25	1		1	
25–29.9	0.83	[0.73–0.96]	1.01	[0.88–1.16]
≥ 30	0.94	[0.78–1.12]	1.07	[0.90–1.28]
Height				
< 160 cm	1		1	
160–169 cm	1.31	[1.08–1.59]	1.32	[1.09–1.61]
170–179 cm	1.44	[1.18–1.76]	1.61	[1.32–1.98]
≥ 180 cm	1.66	[1.21–2.26]	1.73	[1.26–2.38]
Age				
< 25	1		1	
25–29	0.87	[0.77–0.99]	1.04	[0.91–1.19]
30–34	0.82	[0.72–0.94]	0.97	[0.84–1.11]
≥ 35	0.62	[0.51–0.77]	0.68	[0.54–0.84]
Birthweight				
< 3.0 kg	1		1	
3.0–3.5 kg	0.77	[0.66–0.90]	0.66	[0.56–0.78]
3.5–4.0 kg	0.53	[0.46–0.62]	0.49	[0.42–0.58]
> 4.0 kg	0.38	[0.31–0.46]	0.38	[0.30–0.47]
Epidural				
No	1		1	
Yes	0.29	[0.26–0.32]	0.33	[0.29–0.37]
Augmentation with oxytocin:				
No	1		1	
Yes	0.31	[0.28–0.35]	0.40	[0.36–0.45]
Induced labour:				
No	1		1	
Yes	0.91	[0.82–1.02]	1.68	[1.48–1.90]
Phase 1				
	Crude HR	95% CI	Adjusted HR	95% CI
BMI				
< 25	1		1	
25–29.9	0.99	[0.86–1.14]	1.11	[0.96–1.28]
≥ 30	1.01	[0.85–1.21]	1.04	[0.86–1.24]
Phase 2				
	Crude HR	95% CI	Adjusted HR	95% CI
BMI				
< 25	1		1	
25–29.9	0.90	[0.96–1.28]	0.99	[0.86–1.14]
≥ 30	1.06	[0.96–1.28]	1.29	[1.07–1.55]

Phase 1 was defined as the period of time from active labour until the cervix was fully dilated

Phase 2 was defined as the period of time from when the cervix was fully dilated to the actual birth. Caesarean deliveries censored

A hazard ratio (HR) >1 indicates an increased number of vaginal deliveries over time compared to the reference group; a shorter duration of labour. A HR < 1 indicates a decreased number of vaginal deliveries over time compared to the reference group; a longer duration of labour

^a^77 women (4.1%) did not have duration measures recorded and were therefore not accounted for in analysis

The total duration of labour was subdivided into phases. In total, 155 obese women (76.4%) reached phase 2 with full dilation of the orifice, whereas the same applied to 284 (81.1%) overweight and 1112 (89.2%) normal weight women (*p* < 0.01) (Table 4). No significant difference in the duration of phase 1 was found between the BMI groups. However, phase 2 showed a more accelerant course of labour in obese than normal weight women, with a HR of 1.29 (95%CI 1.07–1.55) after adjustment (Table 2).

There were 353 (19.5%) caesarean deliveries in the study popultion comprising 1808 women who had labour duration recorded succesfully. The rate of caesarean deliveries was unequally distributed over the BMI strata, including 16.0% (*n* = 199) of normal weight, 26.3% (*n* = 92) of overweight, and 30.5% (*n* = 62) of obese women being delivered by caesarean (*p* < 0.01). The number of caesarean deliveries was equally distributed between phases 1 and 2 of labour depending on BMI (*p* = 0.30). There was no difference in the use of vacuum extraction.

From logistic regression analysis, we found significantly increased odds ratios (OR) of caesarean delivery with increasing BMI (OR = 1.62, 95%CI 1.18–2.22 among overweight women and OR = 1.76, 95%CI 1.20–2.58 among obese women versus women of normal weight) (Table 4). Adjustments were made for maternal height and age, birth weight, labour induction, epidural analgesia, and administration of oxytocin during labour.

**Fig. 3:**
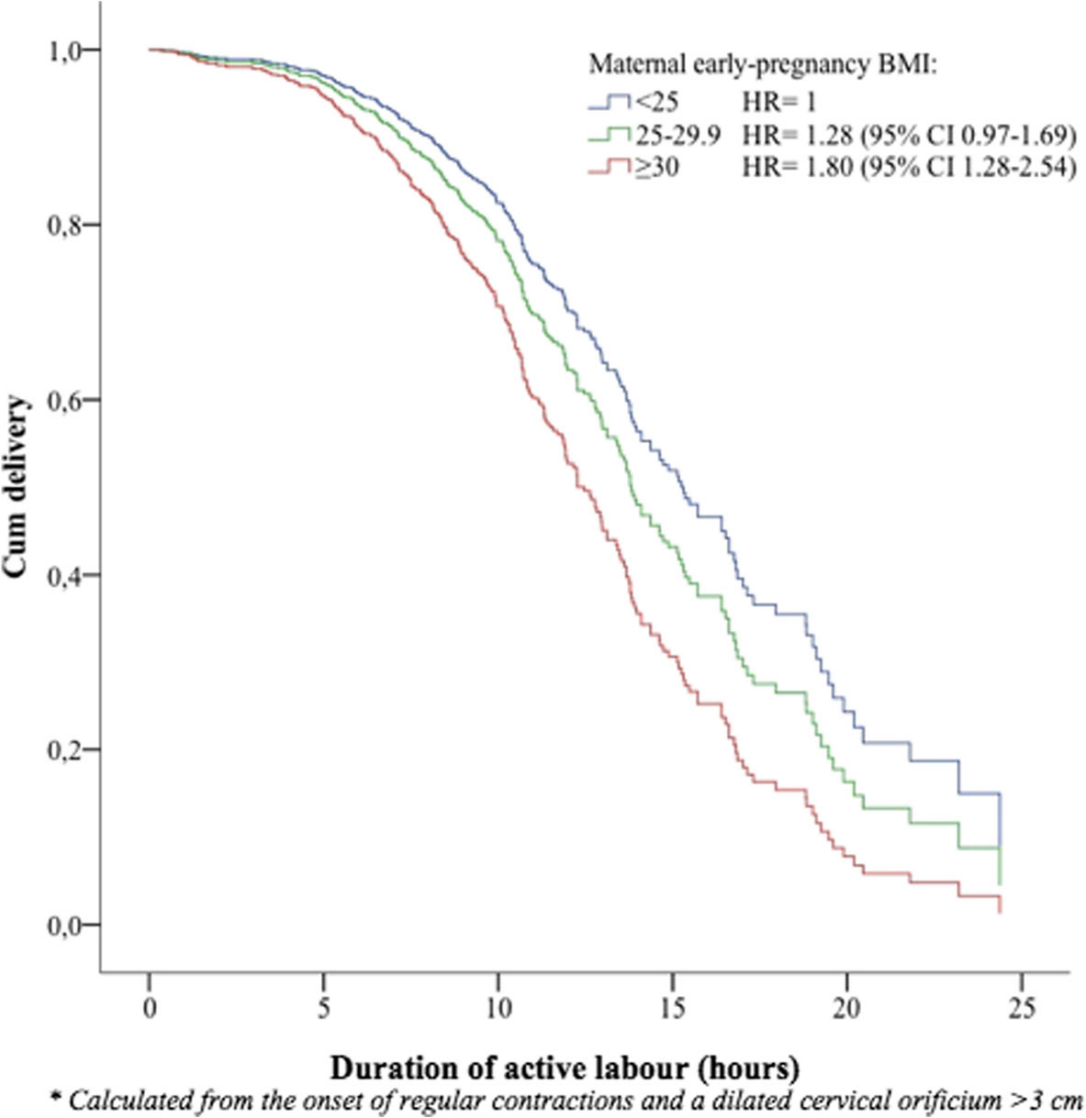
Survival plot as a function of time from the onset of active labour until caesarean delivery. Event was defined as delivery. Women were censored at the time of vaginal delivery. Adjustments were made for maternal age, height, birth weight, labour induction, augmentation with oxytocin, and epidural analgesia. A hazard ratio (HR) >1 illustrates a shorter duration of labour while a HR <1 illustrates a longer duration of labour compared to the reference group consisting of normal-weight women

In univariate Cox analysis, we found an increased HR of caesarean delivery, resulting in a shorter duration from onset of active labour until caesarean with increasing BMI. Compared with normal weight women, the HR of caesarean delivery was 1.39 (95%CI 1.06–1.81) for overweight and 1.94 (95%CI 1.40–2.69) among obese women (Table 3). After adjustment, the results remained significantly increased only for obese women (HR = 1.80, 95%CI 1.28–2.54) (Fig. 3).

**Table 3:** Cox regression showing the hazard ratio (HR) of caesarean delivery comparing the defined BMI groups, with and without adjustment for covariates

	Total labour ^a^			
Covariates	Crude HR	95% CI	Adjusted HR	95% CI
BMI				
< 25	1		1	
25–29.9	1.39	[1.06–1.81]	1.28	[0.97–1.69]
≥ 30	1.94	[1.40–2.69]	1.80	[1.28–2.54]
Height				
< 160 cm	1		1	
160–169 cm	0.83	[0.60–1.15]	0.77	[0.55–1.09]
170–179 cm	0.74	[0.52–1.05]	0.64	[0.44–0.93]
≥ 180 cm	0.67	[0.31–1.41]	0.50	[0.23–1.08]
Age				
< 25	1		1	
25–29	0.99	[0.72–1.37]	0.97	[0.69–1.37]
30–34	1.22	[0.88–1.68]	1.31	[0.94–1.84]
≥ 35	1.57	[1.08–2.29]	1.82	[1.22–2.69]
Birthweight				
< 3.0 kg	1		1	
3.0–3.5 kg	0.89	[0.56–1.41]	0.98	[0.59–1.61]
3.5–4.0 kg	1.03	[0.67–1.59]	1.13	[0.71–1.82]
> 4.0 kg	1.39	[0.87–2.18]	1.43	[0.87–2.36]
Epidural				
No	1		1	
Yes	1.16	[0.88–1.53]	0.96	[0.72–1.29]
Augmentation with oxytocin				
No	1		1	
Yes	1.21	[0.85–1.72]	0.96	[0.65–1.42]
Induced labour				
No	1		1	
Yes	1.80	[1.43–2.27]	1.67	[1.33–2.17]
Phase 1				
	Crude HR	95% CI	Adjusted HR	95% CI
BMI				
< 25	1		1	
25–29.9	1.68	[1.07–2.66]	1.55	[0.97–2.48]
≥ 30	1.56	[0.86–2.84]	1.56	[0.84–2.89]
Phase 2				
	Crude HR	95% CI	Adjusted HR	95% CI
BMI				
< 25	1		1	
25–29.9	1.22	[0.77–1.94]	1.08	[0.67–1.74]
≥ 30	1.56	[0.86–2.84]	1.56	[0.84–2.89]

Phase 1 defined as the period of time from active labour until fully dilated

Phase 2 defined as the period of time from when fully dilated until actual birth. Vaginal deliveries censored

A HR > 1 illustrates an increased number of caesarean deliveries over time compared to the reference group; A shorter duration of labour. A HR < 1 illustrates a decreased number of vaginal deliveries over time compared to the reference group; A longer duration of labour

^a^77 women (4.1%) did not have duration measures recorded and were therefore not accounted for in analysis

Post-partum haemorrhage (PPH) occurred in 122 women (6.5%). The risk of PPH in the obese women was increased compared with normal weight women (OR = 2.04, 95%CI 1.23–2.38). However, after adjustment for birth weight, epidural analgesia, and caesarean delivery, the association was no longer significant (OR = 1.54, 95%CI 0.90–2.61) (Table 4).

**Table 4:** Descriptive and logistic regression analysis regarding secondary outcome variables. Values of odds ratios are presented as crude estimates as well as after adjustment

Secondary outcome variables	*p*-value			Crude OR	95% CI	Adjusted OR	95% CI
Total number of caesarean deliveries ^a^	(*p* < 0.01)	No	Yes				
BMI < 25		1047 (84.0%)	199 (16.0%)	1		1	
BMI 25–29.9		258 (73.7%)	92 (26.3%)	1.88	[1.42–2.49]	1.62	[1.18–2.22]
BMI ≥ 30		141 (69.5%)	62 (30.5%)	2.31	[1.66–3.23]	1.76	[1.20–2.58]
Caesarean prior to onset of active labor ^a^	(*p* < 0.01)	No	Yes				
BMI < 25		22 (1.8%)	1211 (98.2%)	1		1	
BMI 25–29.9		19 (5.4%)	331 (94.6%)	1.99	[1.12–3.52]	1.64	[0.90–2.98]
BMI ≥ 30		18 (8.9%)	185 (91.1%)	3.37	[1.87–6.07]	2.75	[1.47–5.16]
Reached 2nd stage of labour ^a^	(*p* < 0.01)	No	Yes				
BMI < 25		134 (10.8%)	1112 (89.2%)	1		1	
BMI 25–29.9		66 (18.9%)	284 (81.1%)	1.85	[1.38–2.48]	0.61	[0.45–0.84]
BMI ≥ 30		48 (23.6%)	155 (76.4%)	2.13	[1.50–3.03]	0.59	[0.40–0.85]
Vacuum delivery ^b^	(*p* = 0.39)	No	Yes				
BMI < 25		1068 (85.7%)	178 (14.3%)	1		1	
BMI 25–29.9		304 (86.9%)	46 (13.1%)	0.91	[0.64–1.27]	0.83	[0.58–1.18]
BMI ≥ 30		181 (89.2%)	22 (10.8%)	0.73	[0.46–1.17]	0.65	[0.40–1.06]
Post-partum haemorrhage ≥ 1000 mL ^c^	(*p* = 0.02)	No	Yes				
BMI < 25		1176 (89.1%)	70 (10.9%)	1		1	
BMI 25–29.9		325 (92.9%)	25 (7.1%)	1.29	[0.81–2.07]	1.04	[0.64–1.70]
BMI ≥ 30		181 (89.2%)	22 (10.8%)	2.04	[1.23–3.38]	1.54	[0.90–2.61]
Arterial cord pH < 7.05 ^d^	(*p* = 0.04)	No	Yes				
BMI < 25		1128 (98.4%)	18 (1.6%)	1			
BMI 25–29.9		323 (97.9%)	7 (2.1%)	1.36	[0.56–3.28]		
BMI ≥ 30		187 (95.7%)	8 (4.3%)	2.85	[1.22–6.65]		

^a^Adjustments made for: Age, height, birthweight, labour induction, epidural analgesia and use of oxytocin during labour

^b^Adjustments made for: Age and use of oxytocin during labour

^c^Adjustments made for: Birthweight, epidural analgesia and caesarean delivery

^d^No Adjustments were made

There was no difference in neonatal morbidity assessed by Apgar score less than 8 after 5 min and number of admissions to the NICU according to maternal BMI. However, the incidence of an arterial cord pH <7.05 was 4.3% (*n* = 8) in the infants of obese women compared with 2.1% (*n* = 7) in overweight women and 1.6% (*n* = 18) in normal-weight women (*p* = 0.04). The OR of an arterial cord pH <7.05 within the obese group was increased (OR = 2.85, 95%CI 1.22–6.65) compared with the normal-weight group (Table 4).

One neonatal death occurred in the overweight group.

## Discussion

This retrospective cohort study of nulliparous women demonstrated a slight decrease in labour duration during the second phase of active labour, but not in the total duration of active labour, for obese women compared with normal-weight women. Furthermore, caesarean deliveries were performed sooner in obese women following the onset of active labour than in normal-weight women undergoing caesarean, thereby shortening the total duration of active labour in obese women.

A few previous studies suggested no association between labour progression and increasing BMI. A British study of 8350 nulliparous women compared labour progression in obese versus non-obese women, observing no significant difference within the first or second stages of labour [8]. Contrary to our findings, a majority of previous studies report an independent effect of BMI on total duration of active labour. These studies specifically identify the duration of the first stage of labour as being increased, further supporting an overall increase in labour duration [9–11, 13–15]. A study by Kominiarek et al. included 118,978 nulli- and multiparous women in separate analyses. This study found a significant increase in total duration of labour with increasing BMI among nulliparous women [11]. However, the definition of active labour differed from that of most other studies, as a cervical dilatation of only 1 cm was accepted when defining the onset of labour, thereby including what was considered as the latent phase in the current study.

A study by Carlhäll et al. included 63,829 nulliparous women and found a significantly slower progression of labour but reduced duration of the second stage of labour in obese women compared with women with normal BMI [9]. The latter finding is in agreement with that of our study. However, their definition of the second stage of labour was confined to the initiation of pushing efforts and can therefore not be directly compared to the results of our study, as we defined the second stage of labour as the time when the cervical orificium was fully dilated. In contrast to our study, Carlhäll et al. did not include augmentation by oxytocin and the use of epidural analgesia as confounders. Because these parameters have an independent effect on the duration of labour [16] and both appear to be unequally distributed with regard to BMI, this could possibly have distorted the outcomes.

A greater number of censored cases within the obese group during the second stage of labour could have explained our findings of a decreased duration of phase 2. However, this was not the case because caesarean deliveries were less frequently performed in obese women after entering the second stage of labour compared to women of normal BMI. Nevertheless, fewer obese women entered the second stage of labour compared with women of normal weight, thus decreasing the number of women available for the analysis.

We found a significant increase in caesarean deliveries with increasing BMI. This is in accordance with the findings of several larger studies [3–5]. A review by Wispelwey et al. summarized the main risk modulators of caesarean delivery in obese women, including difficulty in initiation of labour and increased induction rates [17]. Since our study only describes women who initiated active labour, and we adjusted for medical induction in statistical analyses it seems likely that there is an independent effect of obesity on the risk of caesarean delivery.

We found that obese women were granted fewer hours of active labour before a caesarean was performed compared with women of normal weight. This could be explained by a possible earlier onset of labour complications within the obese population. However, since there was no difference in the numbers within the different levels of emergency caesareans, this seems unlikely (data not shown). Alternatively, an increased consciousness amongst healthcare staff concerning the issue of maternal obesity may have had an indirect influence on treatment. A more cautious approach to managing these women might have been unknowingly adopted, resulting in an earlier decision to perform a caesarean delivery [18].

The occurrence of PPH >1000 mL was associated with increasing early-pregnancy BMI. In multiple logistic regression analyses, the association was no longer significant, but the estimate still indicated an increased risk of PPH with higher BMI. Accordingly, most other studies found an isolated effect of obesity on the risk of PPH [19].

A slight increase in the incidence of arterial cord pH values <7.05 was associated with increasing early-pregnancy BMI, which could indicate a neonatal outcome that was less positive [20]. The remaining foetal outcome measurements were not associated with maternal BMI.

## Conclusion

We found no association between BMI and the total duration of labour; however, an increased risk of a shorter second stage of labour was apparent for obese women. Our results suggest an overall increased risk of caesarean delivery with increasing BMI. Furthermore, caesarean deliveries performed on obese women were carried out earlier in the course of labour, giving obese women a shorter time of active labour compared with normal-weight women delivering by caesarean.

The risk of PPH in excess of 1000 mL increased with increasing BMI, but only in unadjusted analysis. Additionally, an increased risk of arterial cord blood with pH <7.05 was identified. The remaining neonatal outcome measures were unaffected by BMI.

The results of this study provide valuable information on the expected progression of labour in overweight and obese women and should aid obstetric care providers in deciding if and when to intervene in the labour process. Defining the normal progression of labour for overweight and obese women can help eradicate non-scientific misconceptions about the influence of obesity, resulting in more appropriate treatment of women in this weight group. Finally, the results will aid obstetric health care providers in counselling women on the effect of their overweight.

### Strengths

- Consecutive sampling of women over a 2-year period reduces the selection bias of the cohort
- The sampling of data reveals results that depict everyday practice.
- All charts were manually scrutinized and data for all variables were validated, enhancing the quality of our data
- Adjustments for multiple important confounders were performed.

### Limitations

- The size of the study population.
- BMI was recorded in early pregnancy and this study can therefore not account for the effect of gestational weight gain on labour duration or outcome.
- Gestational age at the first prenatal visit was not recorded; however, most women attended visits early in their pregnancy, as is standard practice
- Measurements of cervical dilation were subjective and were based on examinations performed by numerous midwives
- The results of this study are only applicable to women who reach the active stage of labour as it does not take into consideration the duration of the preliminary latent phase.
- The uniformity of labour management cannot be ensured due to the number of different care providers. However, all personnel are required to follow established guidelines, which should eliminate any substantial differences in the management of care

## Abbreviations

BMI: Body mass index
CI: Confidence interval
CTG: Cartiotocography
HR: Hazard ratio
NICU: Neonatal intensive care unit
OR: Odss ratio
PPH: Post partum haemorrhage
